# Aryl-alkyl-lysines: Novel agents for treatment of *C. difficile* infection

**DOI:** 10.1038/s41598-020-62496-9

**Published:** 2020-03-27

**Authors:** Chandradhish Ghosh, Ahmed AbdelKhalek, Haroon Mohammad, Mohamed N. Seleem, Jayanta Haldar

**Affiliations:** 1 Antimicrobial Research Laboratory, New Chemistry Unit, Jawaharlal Nehru Centre for Advanced Scientific Research, Jakkur, Bengaluru, 560064 Karnataka India; 2 Department of Comparative Pathobiology, Purdue University, 625 Harrison Street, West Lafayette, Indiana 47907 USA; 3 Purdue Institute of Inflammation, Immunology, and Infectious Disease, Purdue University, West Lafayette, IN 47907 USA

**Keywords:** Bacteria, Drug discovery and development

## Abstract

*Clostridium difficile* infections (CDIs) are a growing health concern worldwide. The recalcitrance of *C. difficile* spores to currently available treatments and concomitant virulence of vegetative cells has made it imperative to develop newer modalities of treatment. Aryl-alkyl-lysines have been earlier reported to possess antimicrobial activity against pathogenic bacteria, fungi, and parasites. Their broad spectrum of activity is attributed to their ability to infiltrate microbial membranes. Herein, we report the activity of aryl-alkyl-lysines against *C. difficile* and associated pathogens. The most active compound NCK-10 displayed activity comparable to the clinically-used antibiotic vancomycin. Indeed, against certain *C. difficile* strains, NCK-10 was more active than vancomycin *in vitro*. Additionally, NCK-10 exhibited limited permeation across the intestinal tract as assessed via a Caco-2 bidirectional permeability assay. Overall, the findings suggest aryl-alkyl-lysines warrant further investigation as novel agents to treat CDI.

## Introduction

Colitis or inflammatory bowel disease caused by *Clostridium difficile* is a growing health problem in all parts of the world. *C. difficile* is an endospore forming, Gram-positive anaerobic bacteria that resides in the gastrointestinal tract of humans and animals. A recent study by the U.S. Centers for Disease Control and Prevention (CDC) found that *C. difficile* infections (CDIs) resulted in 29,000 deaths in the USA alone in 2015 and contributed to an annual burden of around $4.8 billion to the healthcare system^1^. Spores of *C. difficile* can survive outside the host intestine and are recalcitrant to heat and standard disinfectants, which make them ideal transmissible agents^2^. The spores, after ingestion, germinate upon exposure to bile salts in the small intestine. These vegetative cells subsequently colonize the colon and release toxins that are responsible for disease symptoms^3^. CDI is often a result of oral antibiotic usage for unrelated infections. Use of antibiotics disturbs the normal microbial flora of the gut allowing *C. difficile* to colonize and promote infection^2,3^.

Although most CDIs can be treated with antibiotics such as metronidazole, vancomycin and fidaxomicin, the spread of hypervirulent strains such as PCR ribotype 027 poses a major challenge^4^. Additionally, relapse of infection is common after cessation of antibiotic treatment^5^. Compounding the challenge further is certain bacterial species that coexist with *C. difficile* in the intestinal tract, such as *Enterococcus*, have already developed resistance to vancomycin. Thus, it might not be long before transmission of resistance to vancomycin is observed in *C. difficile* as well^6^. Furthermore, oral treatment with metronidazole and vancomycin has been shown to promote persistent overgrowth of vancomycin-resistant Enterococci (VRE) during treatment of CDI^7^. In this respect, development of alternative approaches to treat CDI is necessary. Several different approaches are being researched to replace antibiotic treatment including fecal bacteriotherapy, vaccines, and toxin-neutralizing antibodies^8^. Among other strategies, host defense peptides or antimicrobial peptides (AMPs) have attracted significant research efforts in the past two decades as novel antibacterial agents^9^. Several AMPs such as LL-37, NVB-302, surotomycin and ramoplanin have significant activity against *C. difficile* and some of them are also in preclinical studies^10–14^. However, AMPs do possess several challenges to being used as therapeutic agents including susceptibility to degradation by proteases, poor selectivity index and high cost of manufacturing^15,16^. Medicinal chemists have designed several mimics of AMPs which retain their important biological properties while addressing their limitations^17–19^. Indeed two small molecular mimics of AMPs have entered clinical trials for treatment of bacterial skin infections^9^. However, few synthetic mimics of AMPs have been explored for treatment of infections caused by *C. difficile* although the bacterial membrane is considered to be an attractive target for novel antibacterial agents^20,21^.

Aryl-alkyl-lysines were earlier reported as peptidomimetic membrane-active antibacterial agents^22^. They exhibited activity against both Gram-positive and Gram-negative bacteria in animal models of skin infection^23,24^. These molecules also inhibited growth of other pathogens including fungi, parasites and viruses^25–27^. Herein, we report the activity of aryl-alkyl-lysines against clinical isolates of *C. difficile*. Furthermore, the compounds were evaluated for toxicity to human colonic epithelial cells. Finally, Caco-2 permeability assay was conducted with one of the most promising compounds to establish the utility of the compounds for treatment of *C. difficile* infection in colon.

## Results

### Synthesis

**Figure 1:**
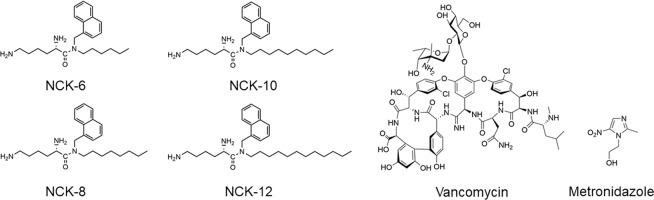
The structures of the compounds used in the study and of clinically relevant antibiotics used as comparators in the study. The long chains have been varied from hexyl (NCK-6) to decyl (NCK-10).

In our studies so far, we have found that the NCK series (consisting of an assembly of naphthalene ring, alkyl chains and L-lysine) of aryl-alkyl-lysine compounds were most active and versatile. Herein, we have conducted the studies with NCK-6, NCK-8, NCK-10 and NCK-12 (Fig. 1). The compounds (with two trifluroacetate counterions) were prepared using a synthetic protocol previously reported^22^. Briefly, 1-naphthaldehyde was reacted with primary alkyl amines (hexyl, octyl, decyl and octadecyl) to form Schiff bases which were further reduced to secondary amines using sodium borohydride. Coupling of these secondary amines with protected Lysine (Boc-Lys(Boc)-OH) and subsequent deprotection of Boc groups yielded the final compounds. These were purified using HPLC before being tested for their biological activity.

### Activity against different strains of *Clostridium difficile*

We had earlier shown that the NCK compounds could infiltrate the membrane of bacteria and also retain activity in acidic pH, other enzymes and in blood^22–24^. We rationalized that because of these properties the NCK compounds would also be active against *C. difficile*. Thus we tested the antibacterial activity of the NCK series of compounds against different strains of *C. difficile* (Table 1). Vancomycin and metronidazole, which are clinically used to treat CDI, were used as comparator drugs. As depicted in Table 2, NCK-6 exhibited poor anticlostridial activity with a minimum inhibitory concentration (MIC) value of 32 µg mL^−1^, while the MIC of NCK-8 ranged from 2 µg mL^−1^ to 8 µg mL^−1^. Both NCK-10 and NCK-12 were quite active against all tested strains of *C. difficile*. The MIC of NCK-10 ranged from ≤0.5 µg mL^−1^ to 2 µg mL^−1^ while that of NCK-12 ranged from 1 µg mL^−1^ to 4 µg mL^−1^, respectively. Against strains P8, P13 and Isolate 10, which were all susceptible to vancomycin (MIC of 0.5 µg mL^−1^) and metronidazole (MIC < 0.25 µg mL^−1^), NCK-10 was active at MICs of 1 µg mL^−1^ to 2 µg mL^−1^. Vancomycin displayed an MIC of 1 and 2 µg mL^−1^ against Isolate 1 and P4, respectively, while NCK-10 was active at 2 µg mL^−1^ against both the strains. NCK-12 was also active against these strains with MIC of 4 µg mL^−1^. NCK-10 displayed most potent activity against strains Isolate 5 and P15 with MIC values less than or equal to 0.5 µg mL^−1^. Against the rest of the strains NCK-10 was active at 1 µg mL^−1^ while the MIC of vancomycin ranged from 0.5 µg mL^−1^ to 1 µg mL^−1^. The broth microdilution assay revealed NCK-10 was comparable to that of vancomycin in inhibiting *C. difficile* growth *in vitro*.

**Table 1:** Source and description of the bacterial strains used in the study.

Bacterial strain	ID-number	Source and description
*C. difficile* Isolate 1	NR-13427	Human patient from the Mid-Atlantic region of the United States in 2008/2009.
*C. difficile* Isolate 2	NR-13428	Human patient from the Mid-Atlantic region of the United States in 2008/2009.
*C. difficile* Isolate 4	NR-13430	Human patient from the Mid-Atlantic region of the United States in 2008/2009.
*C. difficile* Isolate 5	NR-13431	Human patient from the Mid-Atlantic region of the United States in 2008/2009.
*C. difficile* Isolate 6	NR-13432	Human patient from the Mid-Atlantic region of the United States in 2008/2009.
*C. difficile* Isolate 9	NR-13435	Human patient from the Mid-Atlantic region of the United States in 2008/2009.
*C. difficile* Isolate 10	NR-13436	Human patient from the Mid-Atlantic region of the United States in 2008/2009.
*C. difficile* P2	NR-32883	Human patient with CDI in Western Pennsylvania, USA in 2001. Toxigenic strain.
*C. difficile* P4	NR-32889	Human patient with a relapsing CDI in Western Pennsylvania, USA. Toxigenic strain.
*C. difficile* P8	NR-32888	Human patient with CDI in Western Pennsylvania, USA in 2001. Toxigenic strain.
*C. difficile* P13	NR-32891	Human patient with CDI in Western Pennsylvania, USA in 2005. Toxigenic strain.
*C. difficile* P15	NR-32892	Human patient with CDI in Western Pennsylvania, USA in 2005. Toxigenic strain.
*C. difficile* P20	NR-32896	Human patient with a relapsing *C. difficile* infection in Western. Toxigenic strain.
*C. difficile* P21	NR-32897	Human patient with a relapsing *C. difficile* infection in Western. Toxigenic strain.
*C. difficile* P29	NR-32903	Human patient with CDI in Western Pennsylvania, USA in 2009. Toxigenic strain.
*E. faecalis* SF24397	NR-31970	Human urine sample in 2001 in Michigan, USA. Erythromycin- and gentamicin-resistant.
*E. faecalis* B3286	NR-31886	Human blood in 1987 in the United States. Hemolytic isolate with high-level resistance to gentamicin.
*E. faecalis* B3336	NR-31887	Human blood in 1987 in the United States. Shows high-level resistance to gentamicin.
*E. faecalis* B3196	NR-31885	Human blood in 1987 in the United States. Shows high-level resistance to gentamicin.
*E. faecalis* SF28073	NR-31972	Human urine sample obtained in 2003 in Michigan, USA. Resistant to erythromycin, gentamicin and vancomycin.
*E. faecium* E417	HM-965	Human blood in 2006 in Ecuador. Resistant to ampicillin and vancomycin, and displays high levels of resistance to gentamycin and streptomycin.
*E. faecium* UAA714	NR-32065	1994 in Aix-en-Provence, France.
*E. faecium* E1071	NR-28978	Fecal isolate in 2000 in the Netherlands. Non-infectious isolate.

**Table 2:** Antibacterial activity of the aryl-alkyl-lysines, vancomycin, and metronidazole against strains of *Clostridium difficile*.

*C. difficile* (strain)		Minimum inhibitory concentration (µg mL^−1^)					
		NCK-6	NCK-8	NCK-10	NCK-12	Vancomycin	Metronidazole
Isolate 1	NR-13427	64	8	2	4	1	0.25
Isolate 2	NR-13428	64	8	1	2	1	0.0625
Isolate 4	NR-13430	64	8	2	2	0.5	0.5
Isolate 5	NR-13431	32	2	≤0.5	1	0.5	0.25
Isolate 6	NR-13432	64	8	1	2	0.5	0.125
Isolate 9	NR-13435	32	4	1	2	0.5	0.0625
Isolate 10	NR-13436	64	8	1	2	0.5	0.125
P2	NR-32883	64	4	1	1	0.5	0.0625
P4	NR-32889	64	8	2	4	2	0.125
P8	NR-32888	64	8	1	4	0.5	0.125
P13	NR-32891	64	8	2	2	0.5	0.25
P15	NR-32892	64	8	≤0.5	2	0.5	0.0625
P20	NR-32896	64	8	1	2	1	0.25
P21	NR-32897	32	4	1	2	0.5	0.125
P29	NR-32903	32	8	1	2	0.5	0.0625

### Activity against vancomycin-resistant and vancomycin-sensitive *Enterococcus* strains

The NCK compounds were next evaluated against vancomycin-resistant and vancomycin-susceptible strains of *Enterococcus* (Table 3). NCK-6 exhibited poor antibacterial activity against both *E. faecalis* and *E. faecium* (MIC ranged from 64 µg mL^−1^ to 128 µg mL^−1^). The MIC of NCK-8 ranged from 8 µg mL^−1^ to 32 µg mL^−1^ while that of NCK-12 ranged from 4 µg mL^−1^ and 8 µg mL^−1^. All the strains were most susceptible to NCK-10 (MIC ≤ 2 µg mL^−1^). NCK-10 was equally active against both vancomycin-resistant and vancomycin-sensitive strains.

**Table 3:** Antibacterial activity of the NCK compounds and vancomycin against vancomycin-resistant and vancomycin-sensitive strains of *Enterococci*.

Strains	Minimum inhibitory concentration (µg mL^−1^)				
	NCK-6	NCK-8	NCK-10	NCK-12	Vancomycin
*E. faecalis* SF24397	128	16	2	8	1
*E. faecalis* B3286	128	32	2	8	1
*E. faecalis* B3336	128	16	2	8	1
*E. faecalis* B3196	128	16	2	4	1
*E. faecalis* SF28073	128	16	2	4	>128
*E. faecium* E417	64	8	<1	4	>128
*E. faecium* UAA714	128	16	2	8	>128
*E. faecium* E1071	128	16	2	8	>128

### Cytotoxicity of the compounds against human colonic epithelial cells

**Figure 2:**
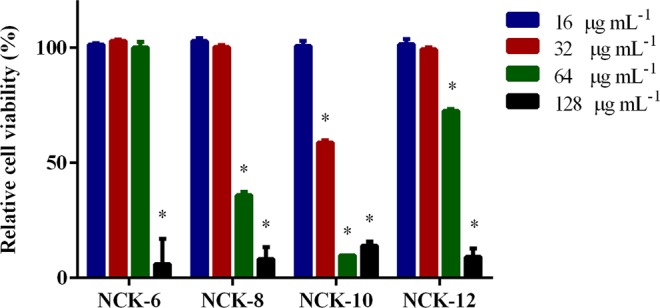
Toxicity of NCK compounds against HRT-18 cell line. *In vitro* cytotoxicity of NCK compounds against human ileocecal colorectal adenocarcinoma (HRT-18) cell line. The toxic effect of NCK compounds was assessed utilizing MTS assay. The viability of the cells was measured after exposure to different concentrations of NCK compounds and was compared to DMSO-treated cells. Asterisk (*) denotes significant difference from DMSO-treated cells using 2-way ANOVA at P < 0.05.

Earlier we had reported that the NCK compounds were selectively active towards bacterial cells^22–24^. Herein, we checked the toxicity of the compounds against human colonic epithelial (HRT-18) cells (Fig. 2). All active NCK compounds were non-toxic at the concentrations that they inhibited the growth of *C. difficile*. The compounds were tested at four different concentrations (128 µg mL^−1^, 64 µg mL^−1^, 32 µg mL^−1^ and 16 µg mL^−1^). It was observed that all compounds were safe to HRT-18 cells at 16 µg mL^−1^. Although the cells survived 32 µg mL^−1^ of NCK-6, NCK-8 and NCK-12, when treated at the same concentration with NCK-10, 60% of the cells survived. Except for NCK-6, some toxicity was observed for all the active compounds at concentrations of 64 µg mL^−1^ and higher. The hemolytic concentration 50 (HC_50_, the concentration at which 50% of human erythrocytes are lysed) of NCK-10 was reported earlier to be 95 µg mL^−1^ ^22^.

### Caco-2 permeability assay

It is important for compounds used to treat CDI to concentrate at the site of infection (in the colon) and not cross the gastrointestinal tract. We thus evaluated if the most potent compound, NCK-10, would permeate across the gastrointestinal tract via a standard Caco-2 bidirectional permeability assay. The assay revealed that NCK-10 (P_app_ <0.01 × 10^−6^ cm s^−1^ from the apical to basolateral compartments) exhibits limited ability to permeate across the Caco-2 monolayer from the apical to basolateral compartment. This result was similar to ranitidine (P_app_ = 0.186 × 10^−6^ cm s^−1^ from the apical to basolateral compartments (Table 4)), a drug known to exhibit limited permeability across the gastrointestinal tract. Ranitidine is typically used as a low-to-moderate permeability control in Caco-2 permeability assays, while warfarin is used as a high permeability control. Talinolol is susceptible to efflux by P-glycoprotein and is thus used as a control drug to determine if a test agent may be susceptible to efflux^28^. The mean apparent permeability values and efflux ratio values obtained for the control drugs were all similar to values presented in previously published reports^28–30^. The results from the Caco-2 bidirectional permeability assay suggest that NCK-10 would concentrate primarily in the gastrointestinal tract which is highly beneficial for treatment of *C. difficile* infections.

**Table 4:** Caco-2 bidirectional permeability analysis for NCK-10 and control drugs.

Test Agent	Mean A → B P_app_(cm s^−**1**^)	Mean B → A P_app_(cm s^−**1**^)	Efflux Ratio (Re)	Notes
Ranitidine	0.186 × 10^−6^	0.942 × 10^−6^	5.18	Low permeability control
Talinolol	0.0272× 10^−6^	2.60 × 10^−6^	97.3	P-glycoprotein control
Warfarin	12.7 × 10^−6^	19.6 × 10^−6^	1.54	High permeability control
NCK-10	<0.01 × 10^−6^	3.26 × 10^−6^	N.D.^a^	Limited permeability

^a^N.D. = not determined (post-assay recovery of NCK-10 was too low from A → B).

## Discussion

*C. difficile* has gained notoriety as the main pathogen responsible for antibiotic-associated diarrhea and nosocomial diarrhea. Moreover, life threatening pseudomembranous colitis and toxic megacolon can also result from *C. difficile*^31^. Although antibiotics are being used to treat CDI, resistance has been reported against metronidazole and treating hypervirulent strains of *C. difficile* is a major difficulty^32^. The scientific community has been trying to use various alternatives to antibiotics as a means to treat CDI including testing antimicrobial peptides and their polymeric mimics^33^. This led us to explore the activity of the aryl-alkyl-lysines against *C. difficile*. Aryl-alkyl-lysines previously were reported to possess good activities against a variety of pathogens both *in vitro* and *in vivo*^23,24^. Ease of synthesis and broad-spectrum activity makes this class of compounds interesting leads as antibacterial agents^22^.

We identified three compounds with moderate to good potency against *C. difficile*. The compound containing a decyl chain was the most active compound (NCK-10). This has been observed against other bacteria and fungi as well. Intriguingly enough, NCK-10 was found to be almost as effective as vancomycin against most strains of *C. difficile* tested with a MIC value of 1 µg mL^−1^ or lower.

A challenge with treating CDI is it leads to overgrowth of *Enterococci*, including strains resistant to vancomycin^7^. Interestingly, we found that NCK-10 was active at 2 µg mL^−1^ and lower against all strains of *Enterococcus* tested, including vancomycin-resistant strains. The NCK compounds were safe to human colonic epithelial cells at concentrations above their MIC against *C. difficile*.

The final point we investigated was the ability of the most potent compound (NCK-10) to cross the gastrointestinal tract. The Caco-2 bidirectional permeability assay is a useful tool in preclinical drug discovery to predict the likelihood of a compound being able to cross the barrier posed by the gastrointestinal tract to gain entry to the bloodstream^34^. For CDI it is important to have compounds that concentrate in the gastrointestinal tract and do not cross into the bloodstream. From the bidirectional permeability assay results obtained, NCK-10 appears unable to effectively cross the gastrointestinal tract. In fact NCK-10 exhibited similar activity to ranitidine, a drug known to exhibit poor permeability across the intestinal tract.

In summary, this study succinctly reports the broad range of activity displayed by the NCK series of compounds against strains of *C. difficile*. Although further studies regarding the mechanism of action of these compounds against *C. difficile* is warranted, previous studies with these compounds suggest they exert their antibacterial effect by disrupting the bacterial membrane^22^. However, the potency displayed by the aryl-alky-lysine compounds indicate further research must be invested towards use of membrane-active small molecules to treat CDI.

## Materials and method

### Antibacterial activity of the compounds against *Clostridium difficile*

The broth microdilution assay was utilized following the Clinical and Laboratory Standards Institute (CLSI) guidelines with slight modifications^35^. Briefly, *C. difficile* strains were grown on anaerobic blood agar plates (Becton Dickinson, BD) and suspended in brain heart infusion supplemented broth (Brain heart infusion medium, BD, supplemented with yeast extract, L-cysteine, Vitamin K_1_ and Hemin, Sigma) to achieve a bacterial concentration of ~10^5^ CFU/ml. The bacterial suspension was seeded in 96-well plates containing drugs/compounds, in duplicate, at the required concentration. Plates were then incubated at 37 °C anaerobically for 48 hours. MICs reported are the lowest concentration of each agent that inhibited the visual growth of bacteria.

### Antibacterial activity of the compounds against Enterococcal species

The broth microdilution assay was used to determine the MIC of the NCK compounds and control antibiotics against different strains of *E. faecalis* and *E. faecium* following the guidelines of the Clinical and Laboratory Standards Institute^36^. Bacteria were incubated aerobically with the NCK compounds or the controls at 37 °C for 16–18 hours before recording the MIC^37–39^.

### Cytotoxicity of the compounds against human ileocecal colorectal adenocarcinoma cell line (HRT-18)

Compounds were assayed at concentrations of 16 µg mL^−1^, 32 µg mL^−1^, 64 µg mL^−1^, and 128 µg mL^−1^ against a human ileocecal colorectal adenocarcinoma cell line (HRT-18) to determine the potential toxic effect to mammalian cells, as described previously^40,41^. Briefly, ~2 × 10^4^ cells suspended in 100 µL of RPMI-1640 supplemented with 10% horse serum were seeded in a 96-well plate and incubated at 37 °C with 5% CO_2_. The cells were cultured for 24 hours until reaching ~90% confluency. The cells were further incubated with the aforementioned concentrations of the compounds for 2 hours. Afterwards, culture media were discarded and the cells in each well were washed with PBS and 100 µL of cell culture media were added prior to addition of the assay reagent MTS 3-(4,5-dimethylthiazol-2-yl)-5-(3-carboxymethoxyphenyl)-2-(4-sulfophenyl)-2H-tetrazolium) (Promega). The plates were incubated for 4 hours at 37 °C in a humidified 5% CO_2_ atmosphere. The absorbance was recorded at 490 nm and the corrected absorbance readings (actual absorbance readings for each treatment subtracted from background absorbance) were taken using a kinetic ELISA microplate reader (SpectraMax i3x, Molecular Devices, Sunnyvale, CA, USA). The quantity of viable cells after treatment with each compound was expressed as a percentage of the control, DMSO. Asterisk (*) denotes a significant difference from the DMSO-treated cells using 2-way analysis of variance (ANOVA) followed by Dunnett’s pairwise comparison.

### Caco-2 permeability assay

The Caco-2 bidirectional permeability assay was conducted as described in previous reports^29,30,41,42^. The Caco-2 cell line is similar to human enterocytes and expresses P-glycoprotein and other relevant metabolic enzymes that affect absorption of orally-administered xenobiotics across the intestinal mucosa^34,43,44^. Caco-2 cells were grown in tissue culture flasks, trypsinized (by rinsing cells with phosphate-buffered saline and then treating with trypsin-EDTA for 5–10 minutes at 37 °C with 5% CO_2_ until cells detached), suspended in Dulbecco’s Modified Eagle Medium with 10% fetal bovine serum, 1% nonessential amino acids and penicillin-streptomycin, and then seeded onto wells of a Millipore 96-well Caco-2 plate. Cells were cultured at 37 °C with 5% CO_2_ until confluent (approximately three weeks) and media was changed at relevant time points. For Apical to Basolateral (A → B) permeability, the test article was added to the apical (A) compartment and the amount of permeation (P_app_, as calculated below) to the basolateral (B) compartment was determined; for Basolateral to Apical (B → A) permeability, the test article was added to the B compartment and the amount of permeation to the A compartment was determined. To test tight junctions and monolayer integrity, the A-side buffer contained 100 µM Lucifer yellow dye in transport buffer (1.98 g/L glucose in 10 mM HEPES, 1 × Hank’s Balanced Salt Solution) at pH 6.5 while the B-side buffer was transport buffer with pH 7.4. Caco-2 cells were incubated with these buffers for two hours, and the receiver side buffer was removed for analysis by LC/MS/MS. To verify the tight junctions and integrity of Caco-2 cell monolayers, aliquots of the cell buffers were analyzed by fluorescence (Lucifer yellow transport ≤ 2%). Any deviations from control values are reported. Data are expressed as permeability (P_app_) = (dQ/dt)/(C_0_A) where dQ/dt is the rate of permeation, C_0_ is the initial concentration of test agent, and A is the area of the monolayer. The efflux ratio (R_e_) was also calculated as follows: R_e_ = (P_app_ B→A)/ (P_app_ A→B); R_e_ > 2 indicates a potential substrate for P-glycoprotein or other active efflux transporter(s).

## Data Availability

All data furnished in the manuscript is available on request.
